# Unexpected low genetic variation in the South American hystricognath rodent *Lagostomus maximus* (Rodentia: Chinchillidae)

**DOI:** 10.1371/journal.pone.0221559

**Published:** 2019-09-12

**Authors:** María Constanza Gariboldi, Pablo Ignacio Felipe Inserra, Sergio Lucero, Mauricio Failla, Sergio Iván Perez, Alfredo Daniel Vitullo

**Affiliations:** 1 Centro de Estudios Biomédicos, Biotecnológicos, Ambientales y Diagnóstico, Universidad Maimónides, Ciudad Autónoma de Buenos Aires, Argentina; 2 Consejo Nacional de Investigaciones Científicas y Técnicas, Ciudad Autónoma de Buenos Aires, Argentina; 3 División de Mastozoología, Museo Argentino de Ciencias Naturales "Bernardino Rivadavia", Ciudad Autónoma de Buenos Aires, Argentina; 4 Proyecto Patagonia Noreste, Río Negro, Argentina; 5 División Antropología, Facultad de Ciencias Naturales y Museo, Universidad Nacional de la Plata, Buenos Aires, Argentina; National Cheng Kung University, TAIWAN

## Abstract

The South American plains vizcacha, *Lagostomus maximus* inhabits primarily the Pampean and adjoining Espinal, Monte and Chaquenean regions of Argentina. In order to study the population genetic structure of *L*. *maximus*, a fragment of 560 bp of the mitochondrial DNA hypervariable region 1from 90 individuals collected from the 3 subspecies and 8 groups along Argentina was amplified and analyzed. We found 9 haplotypes. The haplotype network did not show an apparent phylogeographical signal. Although low levels of genetic variation were found in all the subspecies and groups analyzed, a radiation of *L*. *maximus* would have occurred from the North and Center of the Pampean region toward the rest of its geographic range in Argentina. Low levels of genetic diversity, the existence of a single genetically distinct population in Argentina and changes of its effective size indicate that metapopulation processes and changes in human population dynamics during the late-Holocene were important factors shaping the population genetic structure of *L*. *maximus* in Argentina.

## Introduction

Studying the amount and the pattern of genetic diversity found within and between populations is one of the central aims of population genetics. Furthermore, elucidating the population genetic structure of a species provides ecological and evolutionary information that allows the identification of conservation units. Nevertheless, the population genetic structure of many species is currently poorly understood.

The South American plains vizcacha, *Lagostomus maximus*, is a hystricognath rodent belonging to the family Chinchillidae. The family also comprises the chinchillas (*Chinchilla spp*.) and mountain vizcachas (*Lagidium spp*.). *L*. *maximus* inhabits primarily the Pampean and adjoining Espinal, Monte and Chaquenean regions of Argentina, though it is also found in southeastern Bolivia and western Paraguay [1,2].

Based on morphological characteristics, three subspecies are recognized: *L*. *m*. *petilidens* Hollister, 1914, from southern Buenos Aires, La Pampa and Río Negro provinces (Argentina), *L*. *m*. *maximus* Desmarest, 1817, from central Argentina and *L*. *m*. *immollis* Thomas, 1910, from north-central Argentina, Paraguay and Bolivia [1,3].

*L*. *maximus* is highly social. One to three adult males, two to four times more females and immatures form a social group living in a communal burrow system called "vizcachera" [3,4]. Individuals from each vizcachera share a common home range, with an average size of 1.3 ha, with little overlap between neighboring vizcacheras [4]. Before breeding, young males disperse from their natal burrow system. While adult males move into and out of social groups, not being present in a social group for more than one breeding season, females and inmatures remain in the same group [4,5]. Moreover, *L*. *maximus* is considered an ecosystem engineer, playing important functional roles in grasslands and shrublands. By the construction of burrows and removing the understory vegetation, the species facilitates burrowing owls [6,7]; through grazing, the species changes fire regimen and intensity [8]; furthermore, *L*. *maximus* serves as an important resource for larger predators, such as pumas (*Puma concolor*) [9].

Although many ecological, physiological and anatomical characteristics of *L*. *maximus* have been analyzed (e.g. [1,4,10–12]), no study has investigated the population genetic structure of the species. Considering that *L*. *maximus* presents an extensive distribution in Argentina, that three morphologically distinct subspecies of *L*. *maximus* has been recognized along Argentina [1,3], that females are philopatric and that the average size of the home range of the species is 1.3 ha [4,5], we hypothesize that through the definition of putative geographically distant populations the species in Argentina will display a pattern of population genetic structure showing a significant genetic differentiation among the defined putative populations. To test this hypothesis, we employed mitochondrial DNA (mtDNA) sequences and analyzed the population genetic structure of *L*. *maximus* in Argentina.

## Materials and methods

### Sample collection and DNA extraction

**Fig 1:**
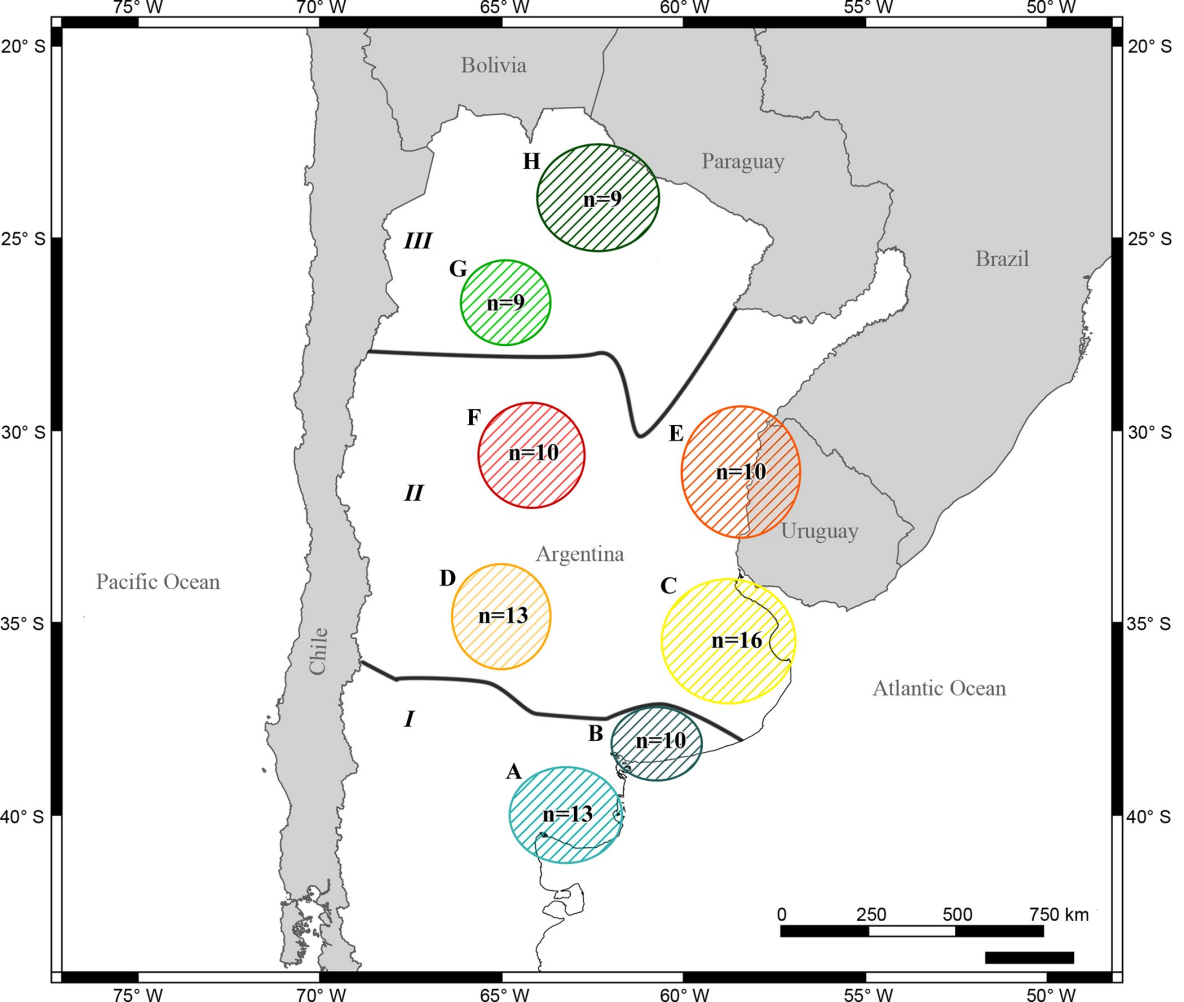
Geographical locations of the sampled sites along the distributional range of *L*. *maximus* in Argentina. I, II and III correspond to *L*. *m*. *petilidens*, *L*. *m*. *maximus* and *L*. *m*. *immollis*, respectively; A-H corresponds to the defined groups in this study. For each group, the sample size is shown.

Samples from 90 individuals were collected along Argentina (Fig 1). As we collected tissue samples from dead individuals or feces, an Institutional Animal Care and Use Committee or equivalent animal ethics committee was not necessary. Sampling permits were issued by Dirección Provincial de Fiscalización Agropecuaria, Alimentaria y de los Recursos Naturales (Buenos Aires Province) and Secretaría de Ambiente y Desarrollo Sustentable (Río Negro Province).

Since samples included bones, muscles and feces, genomic DNA was extracted using different methods. Briefly, bone surfaces were cleaned with 6% sodium hypochlorite and molecular grade water. In order to decalcify each bone, ~0.1g of bone powder was sampled and incubated overnight at room temperature with 1.5 mL 0.5M EDTA pH 8. After incubation, samples were centrifuged at 10,000 rpm for 10 min and the supernatant was removed. To extract DNA from the bone pellet, as well as from fresh muscle, standard proteinase K digestion, phenol-chloroform purification and ethanol DNA precipitation procedures were performed [13]. For stool samples, genomic DNA was extracted using a commercial kit (AccuPrep® Stool DNA Extraction Kit, Bioneer).

In order to perform the analyses, we studied a fragment of the mtDNA control region. This region has been extensively used to investigate the population genetic structure of many species due to its easy collection, lack of recombination and fast rates of base substitution; it has proved to be powerful for evolutionary and genealogical studies (e.g. [14–18]). Moreover, due to the previous recognition of three morphologically distinct subspecies [1,3], the small home range of *L*. *maximus* relative to the extensive analyzed area in this study, the male-based dispersal pattern [4,5], the maternally inheritance of the mtDNA [14,19] and all the above-mentioned characteristics turn the mtDNA control region suitable for studying the population genetic structure of *L*. *maximus*. A fragment of 560 bp of the mtDNA hypervariable region 1 (HVR 1) was amplified by polymerase chain reaction (PCR) using species-specific primers designed for this study: LMF 5' CAA ATC CTG TGT ACT TTG TG and LMR 5' ATG CAT GAC ACC ACA GTT AT. Final concentrations used in PCRs of 25 μl were: 5 μg/ml of template DNA, Buffer 1X (Promega), 0.2 mmol/L of dNTPS, 0.2 μmol/L of each primer, 1.5 mmol/L of MgCl_2_, and 1.25 units of GoTaq polymerase (Promega). PCR cycling profile consisted of an initial denaturation at 94°C for 2 min, followed by thirty-five cycles of denaturation at 94°C for 40 s, anneling at 54°C for 40 s and polymerase extension at 72°C for 40 s, and a final extension at 72°C for 3 min. PCR products were purified with a commercial kit (AccuPrep PCR Purification Kit, Bioneer) and sequenced in both directions using an ABI 337 Automated DNA Prism Sequencer (Applied Biosystems, Inc.).

### Data analysis

CLUSTALX 2.0.11 [20] was used to align DNA sequences and to identify polymorphic sites. Haplotypes were verified using DnaSP v5.10.01 [21].

To study patterns of geographical distribution and haplotype relationships we performed a Median-Joining network [22], as implemented in PopART v1.7 [23].

In order to further evaluate our data set, we grouped the samples by subspecies and geographical proximity (Fig 1). Subspecies division was based on previously published information [1,3] and considering geographical points that share the same elevation. Elevation and distance were assessed using a Geographic Information System (GIS) in ArcGIS software.

For each subspecies and defined group, haplotype (h) and nucleotide diversity (π) of the data set were assessed using Arlequin v3.5 [24]. Additionally, the program MDIV [25], that relies on Markov-chain Monte Carlo (MCMC) simulations, was used to estimate the migration rate per gene per generation between putative populations scaled by the effective population size (M = 2N_e_m). We used the finite sites (HKY) model. Ten independent runs of 2 x 10^6^ iterations each and a burn-in of 5 x 10^5^ iterations were performed. Likelihood values with the highest posterior probability were accepted as the best estimates.

An Analysis of Molecular Variance (AMOVA) was performed to analyze the population genetic structure among the sampling areas using Arlequin v3.5 [24]. Populations pairwise F_ST_ and Φ_ST_ statistics were computed in Arlequin v3.5 [24]. Significance levels (p = 0.05) were assessed using 8,000 nonparametric random permutations and corrected for multiple comparisons with a modified false discovery rate procedure [26] (p = 0.027 for 3 putative populations; p = 0.013 for 8 putative populations).

The historical demography was studied for each of the populations obtained from the population pairwise analysis (see above). A mismatch distribution analysis [27,28] was performed using Arlequin v3.5 [24]. The Harpending’s raggedness index (r) was used to assess the goodness of fit between the observed and expected mismatch [29]. Furthermore, to model *L*. *maximus* demography, and since no substitution rate for the mtDNA control region of the species or a phylogenetically close species has been previously reported, the substitution rate was estimated using BEAST 2.1 [30]. We used the estimated divergence date of Chinchillidae (12.3 million years ago, Mya, 9.3–15.9 Mya) [31]. The HKY model, as indicated by JModelTest [32], a lognormal relaxed clock model, and a Yule speciation model prior was used. The analysis was run for 200 million Markov Chain Monte Carlo (MCMC) steps and the first 10% of runs were discarded as burn-in. Results were checked for convergence to a stationary distribution using Tracer 1.6. Due to the time dependency of molecular evolutionary rates (e.g. [33–35]), the estimated substitution rate was corrected and an order faster substitution rate (7.7 x 10^−7^ substitutions/site/year) was used in this study. A Bayesian skyline plot (BSP) reconstruction was conducted in BEAST 2.1 [30]. Coalescent reconstructions used a strict molecular clock with a substitution rate of 7.7 x 10^−7^ substitutions/site/year, the HKY model of mutation, as indicated by JModelTest [32], and four grouped intervals. Two independent replicates of 200 million MCMC steps each were run. The first 10% of each run was discarded as burn-in. Results were checked for convergence to a stationary distribution using Tracer 1.6 and combined in LogCombiner 1.6.

## Results

From the 560 bp of the HVR 1 analyzed in 90 individuals, 24 variable sites defining 9 haplotypes were found (GenBank accession numbers: MK780072-MK780080).

**Fig 2:**
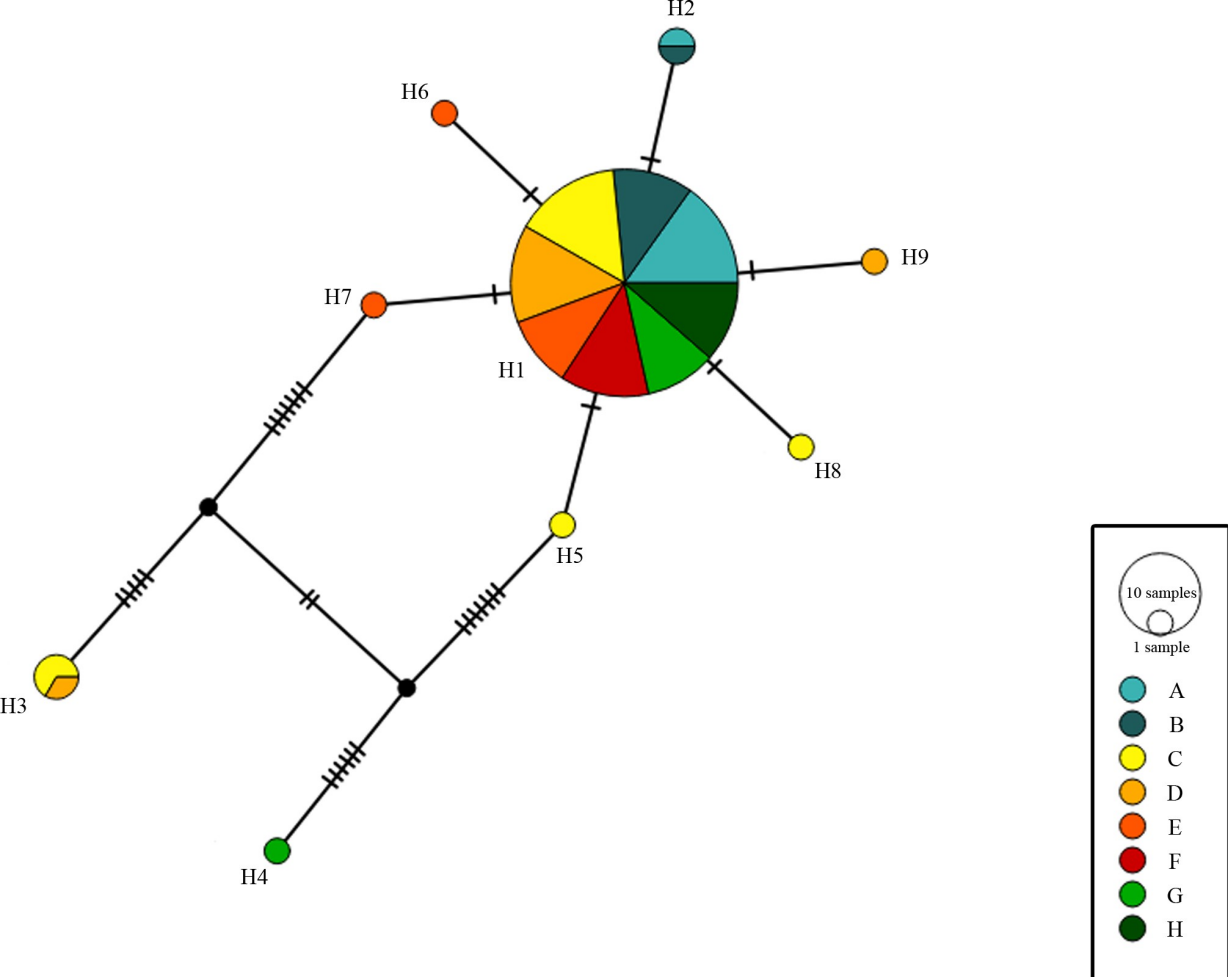
Median-joining network displaying the mtDNA control region variation of *L*. *maximus* in Argentina. Haplotypes are represented with discs and colors that indicate geographical locations. Mutational steps are indicated with stripes. A-H corresponds to the defined groups in this study (see Fig 1).

The phylogenetic analysis based on the Median-Joining network did not reveal a phylogeographic relationship among haplotypes. Haplotype H1 was the most common and widely distributed haplotype, and most other haplotypes connected to it in a star-like topology. However, haplotypes H3 and H4 were separated by 12 and 13 mutational steps, respectively, from their nearest haplotype (Fig 2).

In general, *L*. *maximus* showed a low haplotype and nucleotide diversity (mean 0.200 ± 0.120 and 0.002 ± 0.002, respectively). At the subspecies level, the highest genetic variability was observed in *L*. *m*. *maximus*, while at group level it was observed in group C (Table 1).

**Table 1:** Genetic diversity indexes for each subspecies and group.

Subspecies or group	N	n	H	SD	π	SD
I	23	2	1.66 x 10^−1^	9.76 x 10^−2^	2.96 x 10^−4^	4.63 x 10^−4^
II	49	7	3.00 x 10^−1^	8.48 x 10^−2^	3.08 x 10^−3^	2.03 x 10^−3^
III	18	2	1.11 x 10^−1^	9.64 x 10^−2^	2.78 x 10^−3^	1.95 x 10^−3^
A	13	2	1.54 x 10^−1^	1.26 x 10^−1^	2.75 x 10^−4^	4.59 x 10^−4^
B	10	2	2.00 x 10^−1^	1.54x 10^−1^	3.57 x 10^−4^	5.44 x 10^−4^
C	16	4	4.42 x 10^−1^	1.45 x 10^−1^	5.86 x 10^−3^	3.57 x 10^−3^
D	13	3	2.95 x 10^−1^	1.56 x 10^−1^	3.85 x 10^−3^	2.56 x 10^−3^
E	10	3	3.78 x 10^−1^	1.81 x 10^−1^	7.14 x 10^−4^	8.14 x 10^−4^
F	10	1	0	0	0	0
G	9	2	2.22 x 10^−1^	1.66 X 10^−1^	5.56 x 10^−3^	3.61 x 10^−3^
H	9	1	0	0	0	0

N: sample size; n: number of haplotypes; H: haplotype diversity; π: nucleotide diversity; SD: standard deviation. I, II and III correspond to *L*. *m*. *petilidens*, *L*. *m*. *maximus* and *L*. *m*. *immollis*, respectively; A-H corresponds to the defined groups in this study (see Fig 1).

The AMOVA indicated a non-significant differentiation between the three subspecies (F_CT_ = 0.009, p = 0.255; Φ_CT_ = 0.010, p = 0.256), among groups within each subspecies (F_SC_ = 0.000, p = 0.615; Φ_SC_ = 0.010, p = 0.620) and within groups (F_ST_ = 0.000, p = 0.456; Φ_ST_ = 0.000, p = 0.460). All pairwise comparisons were no significant (p > 0.300 in all cases) (Table 2).

**Table 2:** Pairwise genetic differentiation between putative populations for the mtDNA control region.

	A	B	C	D	E	F	G	H
A	-	0.00	0.04	0.00	0.01	0.00	0.04	0.00
B	0.00	-	0.02	0.00	0.00	0.00	0.00	0.00
C	0.02	0.00	-	0.00	0.01	0.02	0.00	0.01
D	0.00	0.00	0.00	-	0.00	0.00	0.00	0.00
E	0.00	0.00	0.00	0.00	-	0.00	0.00	0.00
F	0.00	0.00	0.07	0.02	0.06	-	0.01	0.00
G	0.00	0.00	0.00	0.00	0.00	0.01	-	0.00
H	0.00	0.00	0.06	0.01	0.04	0.00	0.00	-

A-H corresponds to the defined groups in this study (see Fig 1). Φ_ST_ above diagonal and F_ST_ values below diagonal

In accordance with the population structure results, high levels of gene flow were observed between groups and subspecies (Table 3).

**Table 3:** Estimates of migration rate between groups and subspecies.

		A	B	C	D	E	F	G	I	II
I	A	-							-	
	B	19.9	-							
II	C	16.3	12.0	-					20.8	-
	D	16.1	17.8	16.3	-					
	E	14.0	19.4	16.2	11.3	-				
	F	14.5	14.6	18.7	15.8	10.9	-			
III	G	11.3	19.9	13.5	10.9	10.8	10.2	-	19.1	19.3
	H	14.5	14.6	18.7	15.8	10.9	-	11.9		

I, II and III correspond to *L*. *m*. *petilidens*, *L*. *m*. *maximus* and *L*. *m*. *immollis*, respectively; A-H corresponds to the defined groups in this study (see Fig 1).

**Fig 3:**
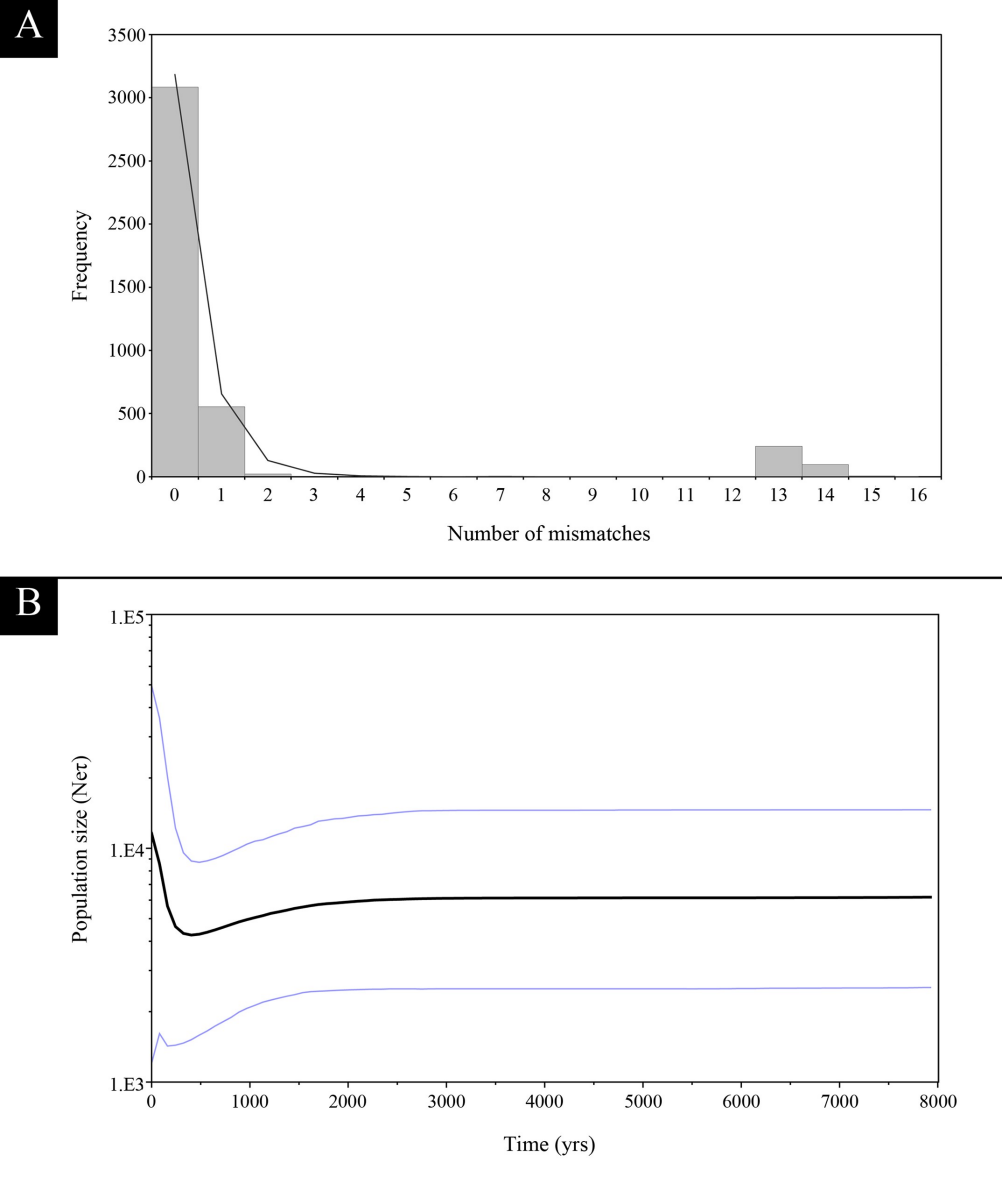
Demographic history based on the mtDNA control region sequences of *Lagostomus maximus*. **(**A) Mismatch distribution. Observed and expected distributions are shown with bars and lines, respectively. (B) Bayesian skyline plot. The black line is the media estimated and the blue lines show the 95% highest posterior density intervals.

Considering a single population, the mismatch distribution tends to show an unimodal L-shaped pattern, suggesting a recent sudden population expansion (Fig 3). Furthermore, the adequacy of the sudden expansion model could not be rejected based on the r (r = 0.42; p = 0.62). The BSP results suggested that the population has kept a stable size until 1,400 years before present (YBP), when a moderately decline in female effective population size began, followed by a population expansion throughout the last 300 years (Fig 3).

## Discussion

### Variability and phylogeography

Despite its extensive distribution along Argentina and neighboring countries, *L*. *maximus* exhibited a low diversity in the mtDNA (Table 1). Haplotype H1 was the most common and widespread along Argentina, occurring in 74 out of 90 individuals (82.22%), and many other haplotypes connected to it in a star-shaped topology (Fig 2). This pattern suggests that haplotype H1 would be an ancestral one from which the others have derived [36]. Moreover, since ancient demographic events usually allow a greater genetic diversification and given the close relationship between the majority of the haplotypes found along Argentina (Fig 2) [37], a relatively recent expansion event would have occurred. Furthermore, even though the genetic diversity levels found in this study were low, the haplotype and nucleotide diversity levels in *L*. *m*. *maximus* were higher, more specifically in groups C and D (i.e., the North and Center of the Pampean region; Table 1). Therefore, considering that older and expanding populations tend to present a higher genetic diversity [38,39], a radiation of *L*. *maximus* would have occurred from this area into the rest of its geographic range in Argentina.

Also, haplotypes H3 and H4 that were found in low frequency were separated from haplotypes H7 and H5 by 12 and 13 mutational steps, respectively (Fig 2). Two plausible explanations could be proposed for the existence of these phylogenetic discontinuities and the apparent lack of geographic separations between these haplotypes and the others found in this study. First, haplotypes H3 and H4 could correspond to nuclear mitochondrial translocations (Numts) (e.g. [40]). However, the use of different tissues, including a mtDNA rich tissue like muscle, and species-specific primers to perform the PCRs, the single band in the electrophoresis gels and the lack of ambiguities in the chromatograms (e.g. [40–42]), turns this possibility less likely. Second, the observed pattern could have arisen from introgressive hybridization between the subspecies of *L*. *maximus* or genetically divergent populations of the species. In fact, introgressive hybridization has been previously observed during range expansions between genetically divergent subspecies or populations not fully isolated (e.g. [43–46]). We have found that haplotype H3 is exclusive from the area where *L*. *maximus maximus* is described, whereas haplotype H4 is exclusive from the area where *L*. *maximus inmollis* is. Therefore, if genetically divergent subspecies or populations existed in those areas and population contractions and expansions occurred (see below), the observed pattern could have arisen.

### Demographic history

During the mid-Holocene (which began 8,000–6,000 years before present, YBP), although some periods and areas of southeastern South America may have presented a humid climate, the climate was characterized by warmer and, primarily, drier conditions than the late-Holocene (that began 3,000–2,000 YBP), and marine transgression-regression that influenced the coastal landscape of several estuaries occurred (e.g. [47–50]).

Climate fluctuations during the mid-Holocene apparently had no significant effect on the population size of *L*. *maximus* (Fig 3). Since *L*. *maximus* occurs in a variety of habitats (from semi-tropical grasslands to desert scrubs) along its range, it would be expected to have the potential to adapt to climate changes. However, this apparent population stability showed in the BSP should be regarded with caution as it could be an artifice of the analysis. Population local contractions and posterior increases can promote the disappearance of some haplotypes lineages, with the subsequent loss of genetic information. In turn, the BSP could display a flat portion preceding a population expansion even though changes in the population effective size of *L*. *maximus* occurred [51,52]. In this respect, during the late-Holocene, a slight population size decline, that started approximately 1,500 YBP, can be observed, followed by its increase after 300 YBP (Fig 3). During the late-Holocene, a change in the human occupation, population density and subsistence strategy began in Argentina (e.g. [47,53,54]). Particularly, the archaeological evidence suggests a rapid increase in the human population size after 1,500 YBP and the increment of human territoriality in several regions [16,53–56]. As the human population size increased, *L*. *maximus* and other small-sized mammals began to be exploited and consumed with more intensity as part of a process of diet expansion or diversification, mainly in the Pampean region [57–59]. This process probably caused a higher predation pressure that could have affected the population size of small-size mammal species. Because *L*. *maximus* and other small-sized mammals were incorporated as secondary preys at that time, the slightly pronounced population size decline of *L*. *maximus* could be directly related with this change in human population dynamics. In the same sense, our demographic results suggest a population size expansion of *L*. *maximus* that began approximately 300 YBP (Fig 3). This demographic expansion is directly related to the decrease in indigenous human population during the Hispanic-indigenous contact period and the changes in the subsistence strategies in Pampa and Patagonia linked to the horse adoption [60].

### Population structure

Typically, phenotypic, behavior and/or genetic changes are often evident along most species range, as a result to their adaptation to heterogeneous environments [61]. Moreover, the genetic changes may vary across the genome as a result of the existence of loci under selection, and those linked to them, and neutral loci [62]. Furthermore, even though neutral regions of the genome may diverge as a consequence of mutation and genetic drift coupled with reduce levels of gene flow, if adaptive divergence increases, neutral gene flow may decrease and a correlation between differentiation at neutral loci and adaptive phenotypic divergence may appear at neutral loci [61,62]. In this study, although along *L*. *maximus* range three subspecies are recognized base on phenotypic changes [1,3], we did not find genetic differences between them (see Results). Factors such as a large population size, high levels of gene flow (but see below) or a recent time since divergence may be preventing us to find genetic differences. When population genetic structure was analyzed considering different groups, we also did not find genetic differences between them (Table 2); furthermore, high levels of gene flow were observed between groups (Table 3). Taking into account the known home range of *L*. *maximus* [4] and the extension of the analyzed area, ongoing gene flow between groups is unlikely. However, if local extinction processes existed in this widely distributed species before and after its population size decline during the late-Holocene that was followed by its expansion after the European contact, the genetic diversity and the genetic differentiation between groups of individuals along the range of this species would be low [38,63,64].

Finally, it is worth to note that although deeper evolutionary processes could have reduced mtDNA diversity and biparental markers variation might yet display a pattern of population structure, the mtDNA is maternally inherited (e.g. [14,19]) and *L*. *maximus* females are philopatric [4,5]. Therefore, it would be expected that the analysis of the mtDNA ought to be the one to allow us to distinguish genetically distant population along Argentina.

### Management and conservation implications

Usually, genetic variation is considered to be critical for the long-term survival of a species. Low levels of genetic variation can affect the ability of a species to respond to stochastic factors (environmental, demographic and genetic stochasticity), as well as to deterministic factors (habitat loss, introduced species and over-exploitation, among others) [65,66]. Additionally, although *L*. *maximus* is classified as "Least Concern" by the International Union for Conservation of Nature [67] and the Sociedad Argentina para el Estudio de los Mamíferos [68], its presence from some areas of Argentina has been reduced mainly due to anthropogenic causes (e.g. [69–72]). The unexpected low levels of genetic variation in *L*. *maximus* in Argentina reported in this study and the anthropogenic pressure to which the species is subjected to in some areas of Argentina should be a matter of concern.

Also, since we found a single genetically distinct population in this study, Argentina can be considered as a single management unit [73]. This provides important information for future reintroduction plans of the species into now-extinct but previously inhabited areas of Argentina. However, in order to assign populations to management units more properly, additional information should be considered, such as life history traits, morphology, habitat and demographic information, as well as genetic data provided by different molecular markers [74,75]. Considering the existence of three morphologically different subspecies, we recommend an averse-to-risk strategy and to consider the three subspecies of *L*. *maximus* as different management units. Furthermore, since the optimal definition of management units and the development of effective management and conservation plans need to be made upon reliable demographic data, demographic studies are crucial in order to ensure the long-term survival of *L*. *maximus*.

## Conclusions

In this study we analyzed for the first time the population genetic structure of *L*. *maximus* along most of its geographic range employing mtDNA. Despite the existence of three morphological distinct subspecies and the wide geographic extension analyzed in this study, our results suggest low levels of genetic diversity in all the analyzed subspecies and groups and a single genetically distinct population along Argentina that displayed changes of its effective size during the last 1,500 YBP. Surprisingly, these results were found by analyzing a genetic marker that is inherited by the philopatric sex. This indicates that different factors, such as metapopulation processes and changes in human population dynamics during the late-Holocene, led to the lack of population genetic structure of *L*. *maximus* in Argentina. Furthermore, since *L*. *maximus* can be considered an ecosystem engineer, our results provide valuable information for future reintroduction plans, especially in degraded areas. Further analyses including highly polymorphic, biparentally inherited microsatellites, as well as ancient DNA from archaeological and paleontological samples, could help to enlarge the results showed herein and improve our knowledge of the population genetic structure at a fine scale and the recent population history of *L*. *maximus*.
